# Tumor immunotherapy: drug-induced neoantigens (xenogenization) and immune checkpoint inhibitors

**DOI:** 10.18632/oncotarget.16335

**Published:** 2017-03-17

**Authors:** Ornella Franzese, Francesco Torino, Maria Pia Fuggetta, Angelo Aquino, Mario Roselli, Enzo Bonmassar, Anna Giuliani, Stefania D’Atri

**Affiliations:** ^1^ Department of Systems Medicine, School of Medicine, University of Rome Tor Vergata, Rome, Italy; ^2^ Department of Systems Medicine, Medical Oncology, University of Rome Tor Vergata, Rome, Italy; ^3^ Institute of Translational Pharmacology, National Council of Research, Rome, Italy; ^4^ Laboratory of Molecular Oncology, Istituto Dermopatico dell’Immacolata-IRCCS, Rome, Italy

**Keywords:** triazene compounds, DNA repair, drug-induced neoantigens, immune checkpoints, cancer immunotherapy

## Abstract

More than 40 years ago, we discovered that novel transplantation antigens can be induced *in vivo* or *in vitro* by treating murine leukemia with dacarbazine. Years later, this phenomenon that we called “Chemical Xenogenization” (CX) and more recently, “Drug-Induced Xenogenization” (DIX), was reproduced by Thierry Boon with a mutagenic/carcinogenic compound (i.e. *N*-methyl-*N’*-nitro-*N*-nitrosoguanidine). In both cases, the molecular bases of DIX rely on mutagenesis induced by methyl adducts to oxygen-6 of DNA guanine. In the present review we illustrate the main DIX-related immune-pharmacodynamic properties of triazene compounds of clinical use (i.e. dacarbazine and temozolomide).

In recent years, tumor immunotherapy has come back to the stage with the discovery of immune checkpoint inhibitors (ICpI) that show an extraordinary immune-enhancing activity. Here we illustrate the salient biochemical features of some of the most interesting ICpI and the up-to-day status of their clinical use. Moreover, we illustrate the literature showing the direct relationship between somatic mutation burden and susceptibility of cancer cells to host’s immune responses.

When DIX was discovered, we were not able to satisfactorily exploit the possible presence of triazene-induced neoantigens in malignant cells since no device was available to adequately enhance host’s immune responses in clinical settings. Today, ICpI show unprecedented efficacy in terms of survival times, especially when elevated mutation load is associated with cancer cells. Therefore, in the future, mutation-dependent neoantigens obtained by appropriate pharmacological intervention appear to disclose a novel approach for enhancing the therapeutic efficacy of ICpI in cancer patients.

## INTRODUCTION

More than 40 years ago we showed, for the first time, that *in vivo* treatment of leukemia bearing mice with the antitumor agent dacarbazine (dimethyltriazene-imidazole-4-carboxamide, DTIC) was able to induce the appearance of novel transplantation antigens (Ags) in malignant cells [1]. This phenomenon was successively termed “chemical xenogenization”(CX) based on analogous definition proposed by Hiroshi Kobayashi in 1969 describing the presence of transplantation Ags induced by Friend virus infection in rat tumors (i.e. “viral xenogenization”, [2]).

After about 15 years of investigations (reviewed in [3]), CX was slowly relegated to oblivion. This was probably due to the lack of instruments that could translate into clinical benefits the appearance of drug-induced neoantigens in patients essentially unable to mount an adequate antitumor immune response.

Few years ago a monoclonal antibody (mAb), Ipilimumab, came to the worldwide attention as a potent inducer of cell-mediated immunity through down-regulation of Cytotoxic T Lymphocyte Antigen-4 (CTLA-4)-mediated T cell suppression [4]. In particular, Ipilimumab was found to substantially increase the survival of patients with advanced melanoma, essentially resistant to classical antitumor drugs. Therefore, on March 25th, 2011 the US Food and Drug Administration approved Ipilimumab for the management of advanced melanoma. This approval was a landmark event in the history of cancer immunotherapy, since for the first time an unusually potent amplifier of T cell-mediated cytotoxic responses was available to oncologists.

This event and the successive appearance in the cancer immunotherapy scenario of a growing number of immune checkpoint inhibitors (ICpI, reviewed in [5, 6]) have provided the ground to bring CX back to life. There is no doubt that drug-induced neoantigens could be considered novel “pharmacologically driven” targets of amplified host's antitumor T-cell responses with great potential therapeutic value.

Up to now, the remarkable progress that has been made in the development of antitumor targeted therapy has not provided a concrete answer to long-term cancer control, especially in solid malignancies. From anti-infective therapy we have learned that, in the absence of adequate host's immune responses, no cure can be attained in spite of the use of insuperably “targeted” agents (e.g. penicillin) in immuno-compromised patients. Therefore, the (re)appearance on the scene of successfully active anti-tumor immunity have disclosed novel and exciting perspectives in cancer management.

## DRUG-INDUCED APPEARANCE OF NON-PREEXISTING TUMOR AGS UNDERLIES CX PHENOMENON

Evidence that *in vivo* treatment with triazene compounds (hereafter referred to as triazenes) including DTIC, is able to induce the appearance of novel transplantations Ags required a long series of investigations.

It was demonstrated that the high doses of DTIC and of the other imidazole or aryltriazenes utilized to induce CX, inhibit severely T-cell dependent graft responses in mice [7]. Therefore, it was necessary to rule out that CX could be due to the emergence of immunogenic sublines in mice immunodepressed by triazenes, and therefore not competent to suppress spontaneously developing immunogenic clones. Two leukemia cell lines were passaged in untreated or DTIC-treated athymic *H-2*d*/H-2*d *nu/nu* BALB/c mice not able to reject allogeneic or xenogeneic cells [8]. In no case, leukemic cells passaged in untreated nude mice became immunogenic for euthymic histocompatible hosts. On the other hand, DTIC treatment of leukemia-bearing nude mice generated highly immunogenic sublines similar to those obtainable in conventional euthymic hosts [8].

In order to consolidate the concept that triazenes induce novel non-preexisting Ags, tolerance studies *in vivo* were performed in BALB/c mice challenged with the Moloney-Leukemia-Virus-induced lymphoma cell line LSTRA, positive for virus-derived Ags. The results showed that mice rendered tolerant to the Ags of the LSTRA cell line, were able to reject DTIC-treated but not untreated LSTRA cells [9].

The final molecular evidence showing that CX is the result of induction of novel Ags was obtained by Grohmann *et al.* in the 1990s. Through an original and highly accurate investigation [10], the authors were able to identify mutated peptides derived from endogenous retroviral *env* sequences detectable in the immunogenic “D” clone originated from xenogenized L5178Y/DTIC cell line. No similar mutated peptides were found in parental, non-xenogenized cells. Transfection experiments showed that products of mutated *env* gp70 subgenic fragments render target cells susceptible to lysis by D-cell primed, *H-2K*d or *H-2L*d-restricted cytotoxic T lymphocytes (CTL, [10]).

In collaboration with Michel Moore's group, D’Atri *et al.* carried out a series of investigations in order to establish whether CX could be induced in human neoplasms [11]. The human lung cancer cell line H-125, treated with an *in vitro* active triazene for a number of cycles, was co-cultured with peripheral blood mononuclear cells of a healthy donor to generate allo-CTL. Thereafter, selected CTL clones able to specifically kill triazene-treated cells but not parental cells were identified. This study supported the hypothesis that CX could be generated also in human tumor cells. However, since no detailed analysis was performed in order to identify possible HLA restriction elements, these results appear to be incomplete and require further investigations.

## KINETICS OF TRIAZENE-INDUCED CX AND IMMUNOGENICITY OF DRUG-TREATED CELLS AT CLONAL LEVEL

**Figure 1:**
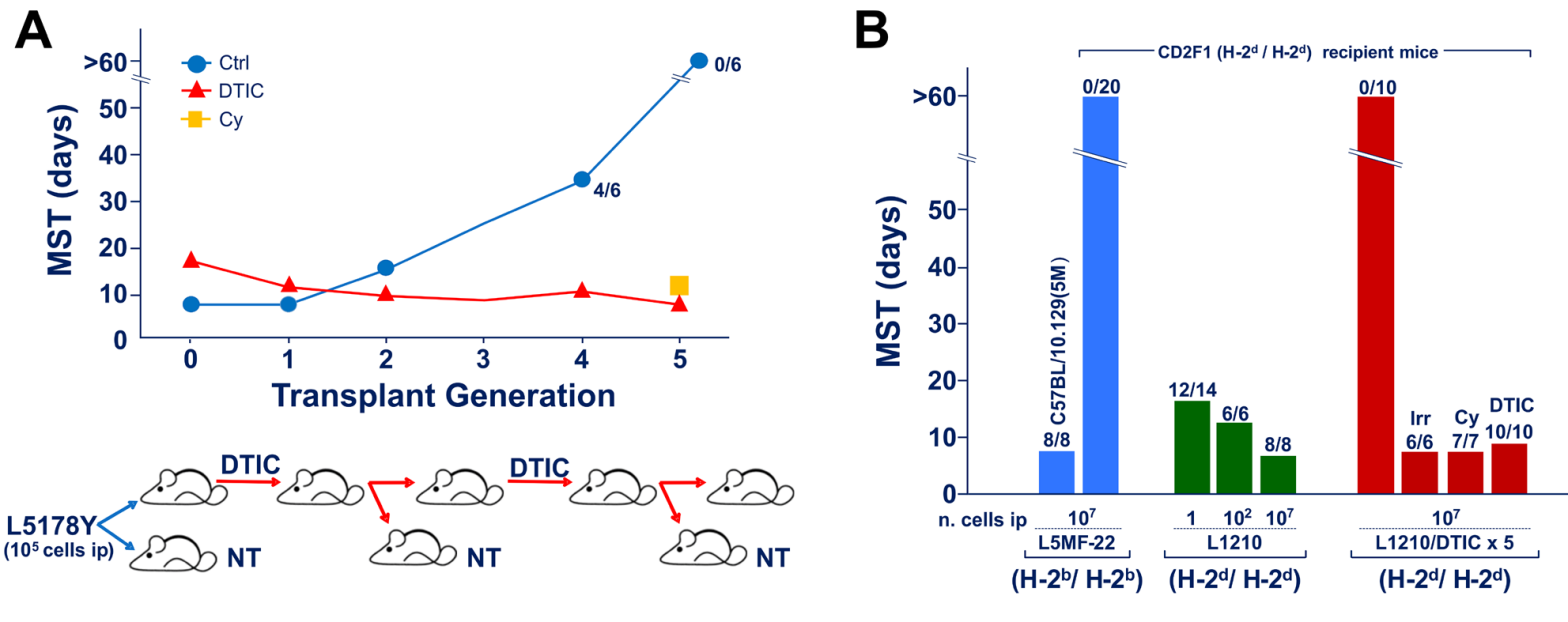
Drug-induced xenogenization (DIX) **Figure 1A**. Typical DIX pattern indicating the kinetics of the appearance of leukemia cell immunogenicity in CD2F1 mice exposed to DTIC (50 mg/Kg/day ip, from day 1 through day 10) after ip challenge with 10^5^ L5178Y leukemia cells of DBA/2 origin. At transplant generation 4, 2 out of 6 control mice not subjected to DTIC treatment, survived longer than the 60 days of observation period. At transplant generation 5, all untreated mice survived beyond the 60 day-observation period. On the other hand, all CD2F1 recipients treated with DTIC or immunodepressed by pre-treatment with cyclophosphamide (Cy, 180 mg/Kg, 6 h before tumor challenge) died with evidence of generalized leukemia at the autopsy. **Figure 1B**. Extent of immunogenicity levels of xenogenized leukemia cells (L1210/DTIC x 5, i.e. L1210 leukemia of DBA/2 origin, treated with DTIC 100 mg/Kg/day for 10 days, for 5 transplant generations). The number of dead mice over the total injected are indicated on the top of each column. Two types of recipient mice were used, i.e. the *H-2*b homozygous C57BL/10.129(5M) mice syngeneic with L5MF-22 leukemia, and the CD2F1 hosts (*H-2*d/*H-2*d), fully histocompatible with L1210 leukemia. The degree of full compatibility between host and tumor is indicated by the observation that almost all CD2F1 mice died for leukemia even after challenge with as low as 1 L1210 cell ip. In addition all C57BL/10.129(5M) mice died with generalized leukemia after injection of 10^7^ cells of the syngeneic L5MF-22 leukemia. The degree of immunogenicity of L1210/DTIC x 5 cells for the H-2-compatible CD2F1 hosts appears to be comparable to that of the H-2-incompatible L5MF-22 for the same CD2F1 recipients. In fact, up to 10^7^ cells of both leukemias were completely rejected by CD2F1 mice that survived beyond the 60 day observation period. On the other hand, all immunodepressed recipients, either irradiated (Irr, 4 Gy delivered on day -1 before challenge), pretreated with cyclophosphamide (Cy, 180 mg/Kg administered 6 h before tumor transplantation) or treated with DTIC (DTIC 100 mg/Kg/day for 10 days) died after challenge with the same number of xenogenized L1210 leukemia cells.

In most of published studies, fully immunogenic xenogenized cell lines were generated following 5-7 transplant generations of treatment with high daily doses of triazenes (see Figure 1A). The magnitude of graft response of histocompatible mice against triazene-treated cells was found to be comparable to that detectable in mice challenged with major histocompatibility complex (MHC)-incompatible malignant cells [12]. Actually, the status of “fully immunogenic” xenogenized cells is revealed by the rejection of at least 10^5^ (and sometimes up to 10^7^) triazene-treated cells by intact, wholly histocompatible recipients. It is noteworthy that in the same host/tumor systems, even 1 murine leukemia cell (e.g. L1210 leukemia in DBA/2 or CD2F1 mice) is often able to kill the untreated host with generalized leukemia within 15-17 days (see Figure 1B).

A typical kinetics of the xenogenization process occurring in CD2F1 mice challenged with L5178Y leukemia (10^5^ cells ip) and treated with DTIC [13] is illustrated in Figure 1A. At transplant generation “0” (i.e. at the beginning of the process), DTIC-treated mice showed a median survival time (MST) longer compared to controls, although no animal survived beyond the 60-day observation period. At transplant generation 1 and 2 no significant difference in MSTs was noticed between control and DTIC-treated mice, probably indicating the onset of drug resistance in leukemic cells exposed *in vivo* to the alkylating agent. Progressively, from transplant generation 3 onward, control mice survived significantly longer than DTIC-treated recipients, and at transplant generation 5 all controls were long-term survivors, whereas all DTIC treated mice died, with an MST of 10 days. This phenomenon was interpreted as a result of host's graft response against highly immunogenic DTIC-treated cells. Indeed, intact mice rejected the tumor, whereas DTIC-treated hosts, which were severely immunodepressed by the compound [7, 14], or mice pretreated with cyclophosphamide succumbed with generalized leukemia. This finding ruled out the possibility that DTIC-treated L5178Y was a leukemia subline dependent on DTIC for growth.

Similar results were obtained with a number of mouse leukemias and the degree of immunogenicity of drug-treated cells for the histocompatible host was often similar to that detectable in target cells incompatible for the entire H-2 haplotype. In fact, the results illustrated in Figure 1B show that all intact CD2F1 mice that were able to reject 10^7^ cells of the H-2-incompatible L5MF-22 leukemia, were also able to reject 10^7^ cells of the histocompatible L1210 cells subjected to 5 transplant generations of DTIC treatment. On the other hand, all mice immunodepressed by total-body irradiation, or by pretreatment with DTIC or cyclophosphamide succumbed with generalized leukemia following challenge with the “xenogenized” L1210 leukemia cells.

Of particular interest are the findings that CX is also inducible *in vitro* [15] and that the highly immunogenic triazene-treated cells obtained either *in vivo* or *in vitro,* retain their immunogenic properties after up to 90 passages in immunodepressed mice not exposed to triazenes [13]. It is obvious that these tumor neoantigens are heritable after a number of malignant cell divisions and can thus be considered immunological targets even after triazene withdrawal. Therefore the presence of drug-induced neoantigens in tumor cell population could be of therapeutic advantage for different immunotherapeutic strategies in cancer treatment.

**Figure 2:**
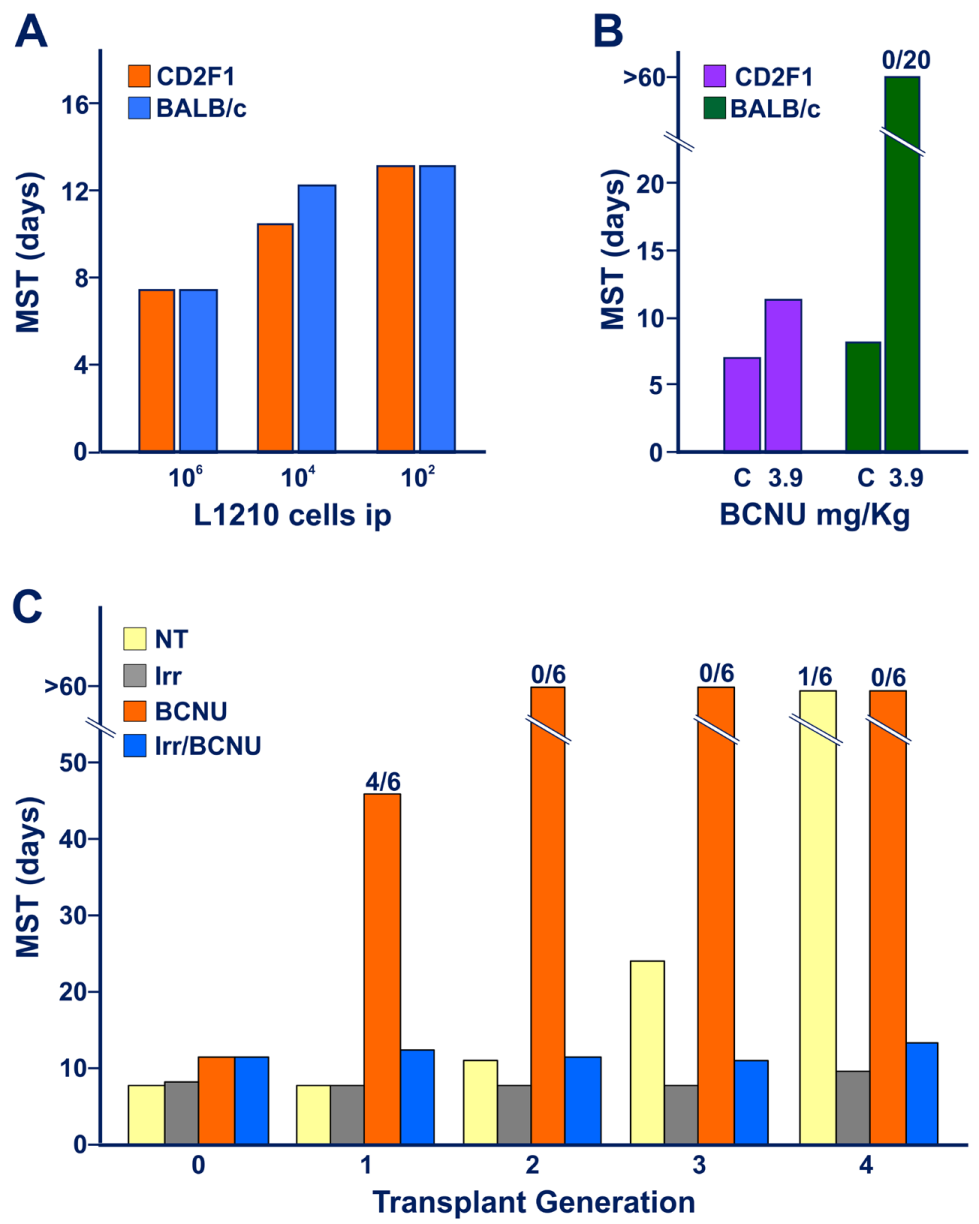
Appearance of limited degree of immunogenicity in L5178Y DBA/2 leukemia cells exposed to early transplant generations of DTIC treatment, revealed by immuno-chemotherapy synergism When not specified in terms of number of dead mice over the total tested indicated on the top of the columns, all mice (6-8 animals for group) died with generalized leukemia. **Figure 2A**. Graded numbers of L1210 leukemia cells were inoculated into fully histocompatible CD2F1 mice, or into *H-2*d-compatible BALB/c mice, incompatible for multiple minor histocompatibility antigens (see Ref 17). The marginal allograft response of BALB/c hosts was not adequate to restrain the growth of leukemic cells, as evidenced by the finding that no substantial difference in median survival time was detected between CD2F1 and BALB/c mice inoculated with as low as 10^2^ L1210 cells ip. **Figure 2B**. CD2F1 and BALB/c mice were inoculated with 10^5^ L1210 cells ip. In this case, CD2F1 mice treated with a low dose of BCNU, (3.9 mg/Kg ip, administered on day 3 after tumor transplantation) showed a limited increase in MST with respect to that of untreated controls (C). In contrast, all BCNU-treated allogeneic BALB/c mice, survived beyond the 60 day-observation period, thus confirming the possibility of revealing a marginal antitumor graft response of the host through an immune-chemotherapy synergistic effect. **Figure 2C**. (data from Ref 13). The strategy of immune-chemotherapy synergism indicates that murine leukemia cells exposed *in vivo* to DTIC acquire appreciable levels of immunogenicity already at transplant generation “1”. Malignant cell immunogenicity progressively increases at the successive generations of treatment with the triazene compound. This figure illustrates the results of a typical experiment performed to evaluate the immunogenic properties of L5178Y leukemia cells in the course of the first 4 transplant generations of DTIC treatment (DTIC 100 mg/Kg/day ip for 10 days) in CD2F1 mice. Blasts (10^5^ cells) obtained from non-treated leukemic donors (i.e. at transplant generation “0”) or from DTIC-treated leukemic donors (at transplant generations 1 through 4) were inoculated into 4 groups of mice, i.e. non-treated (NT), immunodepressed through exposure to total-body irradiation (Irr, 4 Gy X rays, on day -1), treated with BCNU (10 mg/Kg ip), immunodepressed (i.e. pre-irradiated) and treated with BCNU (Irr/BCNU). An additional group of mice was treated with DTIC to obtain a further generation of treatment with the triazene compound. At transplant generation “0” the intact L5178Y cells did not show appreciable immunogenicity, since all non-immunodepressed or irradiated recipients treated with BCNU showed a similar modest increase of MST over that of non-treated controls. Remarkably, at transplant generation “1” instead, L5178Y cells obtained from DTIC-treated donors showed immunogenicity strength similar to that conferred by products of minor histocompatibility loci. This is evidenced by the consistent increase of survival times of BCNU-treated animals respect to those of mice not subjected to chemotherapy, or treated with BCNU but immunodepressed by means of total-body irradiation.

Further experiments were conducted in order to explore whether a limited degree of immunogenicity could be revealed during the initial 1-3 transplant generations of triazene treatment using a protocol based on immuno-chemotherapy synergism [16]. Figures 2A and 2B show the survival times of mice challenged with L1210 leukemia of DBA/2 origin. No difference in survival times was detected between fully histocompatible CD2F1 mice and *H-2*d-compatible BALB/c mice incompatible for minor histocompatibility loci. However, while treatment with a low dose of bis-chloroethyl-nitrosourea (BCNU) was minimally active in CD2F1 hosts, in the majority of experiments, it was able to “cure”, all allogeneic BALB/c mice [17] that are thought to be able to mount a weak allograft response. In this model, therefore, synergism between weak graft response and chemotherapy reveals antitumor immune reactions not easily detectable without drug treatment.

**Figure 3:**
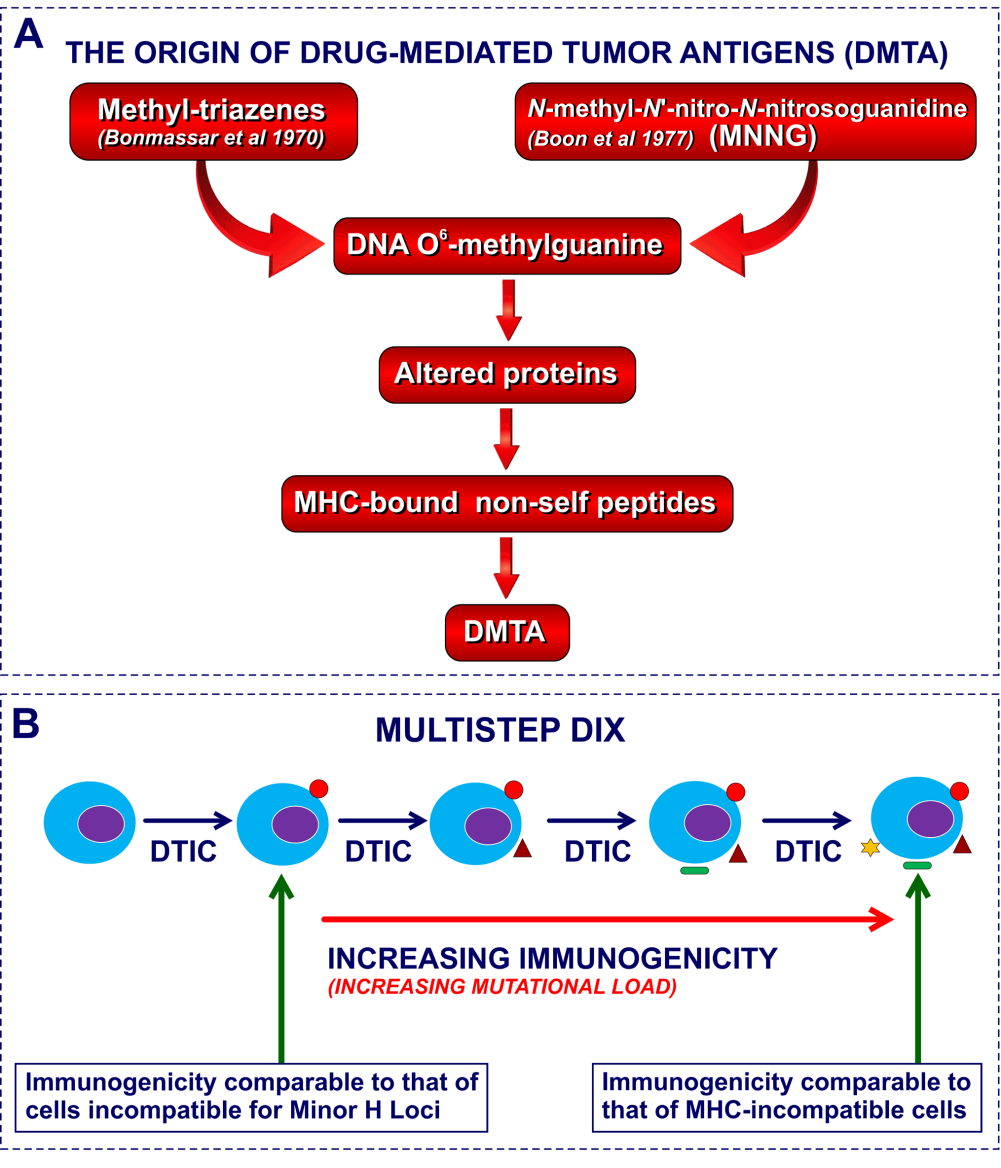
Molecular basis of DIX and CX: DNA O 6-methyl-guanine adducts. **Figure 3A**. The mutation-dependent origin of DIX and CX. DMTA, drug-mediated tumor antigens, i.e neoantigens induced by drug-treatment. **Figure 3B**. The hypothesis of increased mutational load as the mechanism underlying the progressive increase of immunogenicity in the course of sequential transplant generations of DTIC treatment

As illustrated in Figure 2C, CD2F1 mice were inoculated with leukemic cells obtained from DTIC-treated donors during initial transplant generations of DTIC treatment, before the appearance of strong immunogenicity in L5178Y/DTIC leukemia. Animals were then treated with low-dose BCNU and survival time analysis showed longer overall survival than those subjected to the same treatment, but immunodepressed with total-body irradiation delivered one day before tumor challenge. This observation suggests a stepwise increase in immunogenicity of leukemic blasts exposed *in vivo* to daily pulses of DTIC, possibly as a result of a progressive rise of mutation load, in line with the hypothesis illustrated in Figure 3 (see below).

The appearance of relatively weak immunogenicity after a single exposure to DTIC was confirmed in CD2F1 mice inoculated ip with 10^8^ L1210 cells and injected 1 h later with a single high-dose of DTIC (300 mg/Kg, [18]). Again, almost all intact CD2F1 mice challenged with 10^6^ leukemic cells collected from the DTIC-treated donors were “cured” by a single dose of BCNU (10 mg/Kg), whereas most recipient mice exposed to the same treatment, but immunodepressed with cyclophosphamide, died with generalized leukemia.

After the discovery of CX [1], in view of its possible biological and clinical relevance, some fundamental cellular and molecular aspects of the xenogenization process were investigated by different groups. Great priority was given to studies aimed at establishing whether different xenogenized neoplastic cells present cross-reacting neoantigens, or if each triazene-treated tumor is composed of a homogeneous cell population containing a single set of neoantigens, or of a number of clones showing different non-cross-reacting neoantigens. In 1986 the group of Angelo Nicolin [19] cloned a xenogenized L1210 leukemia cell line and analyzed the single clones for immunogenicity and cross-reactivity in terms of CTL generation and susceptibility to lysis. They concluded that the xenogenized leukemia contained a heterogeneous but limited number of different antigenically cross-reacting and non-cross reacting cell clones. Later, Marelli *et al*.[20] xenogenized a homogeneous mouse leukemia cell population starting from a single L1210 clone, cloned the DTIC-treated cell line and evaluated the immunogenic properties and cross-reactivity of the clones. The authors concluded that at least one common specific neoantigen is reproducibly elicited by DTIC treatment within an identical malignant cell population. In the light of the mutational mechanism underlying CX (see below), it is reasonable to hypothesize that multiple point mutational events generated by a triazene-targeted hot spot could result in the appearance of common non-self peptide(s) in leukemic cell population. Notably, no CX affecting normal bone marrow cells was detected in DTIC-treated mice (Bonmassar E *et al*., unpublished data).

## CX IS THE RESULT OF DRUG-INDUCED MUTATIONAL MECHANISMS LEADING TO THE APPEARANCE OF NON-SELF IMMUNOGENIC PEPTIDES

The first report describing CX [1] already contained the hypothesis that this phenomenon could have been generated by somatic mutations, since DTIC was classified as a carcinogenic compound able to alkylate DNA [21, 22]. Further studies established that Quinacrine, an antimalarial drug with anti-mutagenic activity [23], suppresses entirely CX without impairing the antitumor and the immunosuppressant activity of DTIC [24]. However, direct evidence that a mutational mechanism was involved in triazene-induced appearance of neoantigens was not obtained until the molecular investigations performed by Grohmann *et al.* in the 1990s [10, 25, 26]. As previously mentioned, the authors found that CX was the result of point mutations provoked mainly by triazene-induced methyl adducts to the oxygen 6 of DNA guanine, affecting retroviral sequences normally present in mouse genome [27], followed by the appearance of MHC-restricted highly immunogenic non-self peptides.

Seven years after the discovery of DTIC-induced CX, the group of Thierry Boon found that selected clones obtained from a mouse teratocarcinoma cell line treated *in vitro* with the mutagen/carcinogen *N-*methyl-*N*’-nitro-*N-*nitrosoguanidine (MNNG) were rejected by histocompatible recipients through an immunomediated mechanism [28]. MNNG is a classical mutagen that adds methyl groups to a number of nucleophilic sites on DNA bases, including the oxygen-6 of guanine [29]. In particular, if not repaired (see below), the O^6^-methylguanine (O^6^-MeG) is responsible of point mutations leading to the appearance of specific Ags able to elicit cell-mediated immune responses and graft rejection. Boon and his group identified several MNNG-treated tumor cell sublines that were termed *tum*- *variants* since they were unable to grow into immunocompetent histocompatible mice, but grew rapidly in immunodepressed recipients and elicited cytotoxic T-cell responses [28, 30]. In addition, the mutational mechanism underlying the biological features of *tum*- *variants* was confirmed by molecular investigations of the same group between 1988 and 1990 [31, 32]. In a mouse mastocytoma P815 model they identified genome sequences targets of MNNG-induced mutations, responsible for malignant cell immunogenicity. In the meantime, Altevogt *et al*. [33] found that a similar mechanism underlies the immunogenicity of the mouse lymphoma cell line Eb (a subline of L5178YE) exposed to MNNG. Also in MNNG-induced xenogenization, tumor cell immunogenicity was based on the presence of mutation-generated MHC-restricted non-self peptides [34].

All these findings indicate that malignant cell xenogenization can be obtained with two distinct classes of compounds, i.e. triazenes of large clinical application and MNNG, a mutagenic compound of no use in cancer chemotherapy (see Figure 3). Therefore, in the present review we have decided to maintain the term “CX” for mutagen-induced xenogenization and to adopt the new term “Drug-Induced Xenogenization” (DIX) to describe the induction of novel antigenic specificities by exposure to pharmacological agents.

## NEOANTIGENS GENERATED BY DIX ELICIT CELL-MEDIATED AND HUMORAL IMMUNITY *IN VIVO* AND *IN VITRO*

Immune responses against xenogenized leukemia cells were found to be extremely complex, including T-cell dependent graft responses (as detailed in the previous sections), T-cell independent, radio-resistant graft response [35], H-2-restricted T-cell mediated cytotoxicity [10, 19, 20, 25, 26, 36–38] and weak humoral responses [39–43]. The T-cell dependency of the classical graft rejection found in histocompatible mice was confirmed by the finding that BALB/c *nu/nu* mice were not competent for rejecting xenogenized leukemia cells [8]. In addition, the growth kinetics and rejection pattern of xenogenized cells in the peritoneal cavity of mice were similar to those of untreated leukemic cells transplanted into H-2-incompatible recipients [44]. Significant impairment of xenogenized blast growth limited to the splenic territory, was found in lethally-irradiated histocompatible mice [35], suggesting that triazene-treated cells could be susceptible to radioresistant natural immunity of “hybrid resistance (Hh)” type [45, 46].

## DIX/CX AND ROLE OF DNA REPAIR ENZYMES

**Figure 4:**
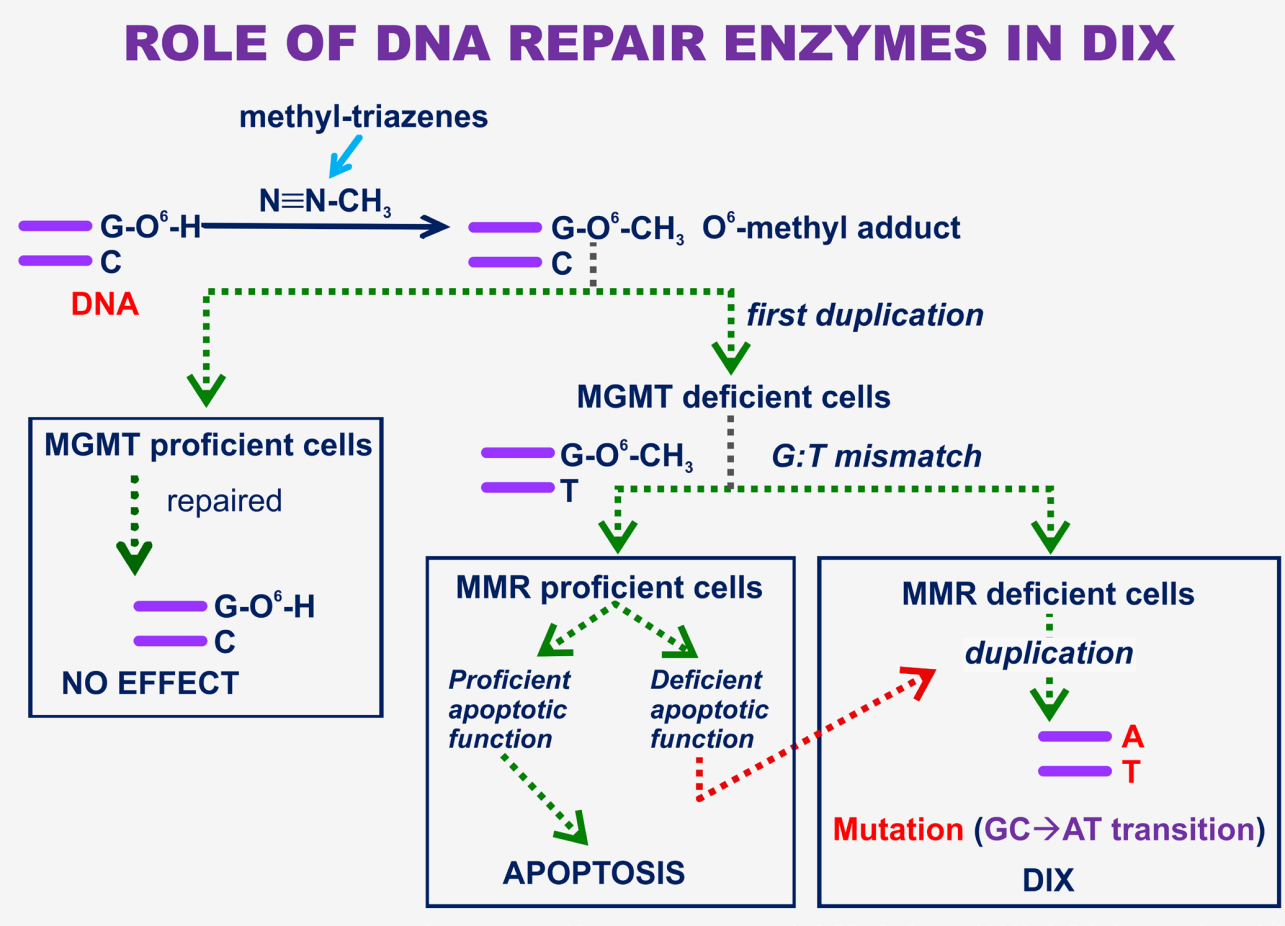
The role of DNA repair enzymes in DIX The end-metabolic product of triazene compounds of clinical use (i.e. DTIC and temozolomide) is diazomethane [46] that reacts with the oxygen-6 of DNA guanine, generating an O^6^-methyl adduct. If target tumor cells express high levels of the DNA repair MGMT that acts as methyl acceptor molecule, all adducts are rapidly repaired and triazene treatment is totally ineffective. On the other hand, if MGMT levels are low or are down-regulated by MGMT inhibitors (e.g. Lomeguatrib, [202]), the adduct is stable and a mismatch signal is generated by C:O^6^-MeG, and after one duplication by frequent G:T mismatches. If the cells express a fully active MMR system and the apoptotic function is not compromised, triazene treatment results in target cell apoptotic death. Alternatively, if neoplastic cells are MMR deficient or their apoptotic pathways do not work properly, malignant growth normally goes on and mutation of G:C -> A:T transition type frequently occurs, leading to DIX appearance.

The antitumor activity and the DIX property of triazenes are crucially dependent on the function of different DNA repair enzymes (reviewed in [47] and briefly summarized in Figure 4). In particular, triazenes and MNNG are mutagenic since they both induce methyl adducts to the oxygen-6 of DNA guanine (reviewed in [48]), This type of biochemical lesion is also responsible for the antitumor activity of triazenes as evidenced by Catapano *et al*. [49]. The cytotoxic and xenogenizing activity of these DNA mono-methylating compounds is antagonized by the DNA repair enzyme O^6^-methylguanine-DNA methyltransferase (MGMT). If the drug-treated cell does not eliminate the adducts through adequate nuclear levels of MGMT, O^6^-MeG of DNA loses pairing compatibility with C and triggers a Mismatch Repair (MMR) response [47, 50]. Since the MMR system is competent to replace an appropriate nucleotide in the newly formed strand but not in the “old” strand during DNA synthesis, no repair by this enzymatic system is feasible. It follows that after several “futile” unsuccessful repair attempts, apoptotic signal can be generated and, if the apoptosis machinery is correctly working, the damaged cell dies. In the absence of a functional MMR system, proliferation is not impaired. Frequent G:C to A:T transitions occur since during the DNA duplication process O^6^-MeG preferably pairs with T rather than with C, and at the second round of duplication, the new strand will contain A instead of G (Figure 4). Therefore, the molecular mainstay of DIX relies on MGMT and MMR deficiency that assures the highest mutation load attainable following triazene treatment. In contrast, the cytotoxic activity of these agents requires low levels of MGMT but a fully efficient MMR system [47]. This seems to be in line with previous findings illustrated by Fioretti *et al.* [51] that demonstrated a clear dissociation between the onset of resistance to DTIC treatment and emergence of DIX, although no data on MMR status of target leukemia cells was mentioned in that report.

## PRECLINICAL STUDIES ON DIX-MEDIATED ANTITUMOR IMMUNO-CHEMOTHERAPY

Since host's antitumor responses appear to be mandatory for a successful treatment of cancer, numerous attempts have been made to exploit DIX for treatment of primary recipient mice bearing a non-treated parental leukemia. Therefore, histocompatible mice bearing L1210 were treated with a xenogenizing and immunosuppressive dose of DTIC (50 mg/Kg/day for 5 days) to induce a limited but significant DIX. Thereafter, recipient mice were infused with intact syngeneic splenocytes to restore immune reactivity compromised by DTIC administration, followed by treatment with BCNU in order to obtain synergistic effects between host's graft response against xenogenized leukemia and chemotherapy. However, this protocol did not work until it was modified by adding a single limited dose of cyclophosphamide (100 mg/Kg) administered before spleen cell injection [52]. In this case a relatively high number of long-term survivors was observed, demonstrating in a preclinical model that DIX strategy can be a realistic approach for cancer immuno-chemotherapy. The role of cyclophosphamide in this study has not been definitely established although it could be ascribed to regulatory T cell (Treg) suppression and/or dendritic cell maturation [53, 54]. In any case, several antitumor agents, including cyclophosphamide and irradiation have shown a number of immunoenhancing effects (reviewed in [55]). In a mouse model, cyclophosphamide produces an initial lymphoid organ depletion which enhances the activity of adoptively transferred antitumor immune cells. This effect appears to be driven mainly by a cyclophosphamide-induced “cytokine storm” within 48 h from the injection [56], consisting in the up-regulation of interleukin (IL)-1, IL-2, IL-7 and IL-21, essential for T cell homeostatic proliferation.

Consistent with these findings, a phase I/II clinical trial, performed on 10 disease-free HLA-A2^+^ melanoma patients, has proven that vaccination with Melan-A and gp100 peptides in combination with DTIC (administered one day before vaccine inoculation) results in an improved cellular immune response as compared with vaccination alone and prevents melanoma relapse [57]. Global transcriptional analysis of peripheral blood mononuclear cells revealed a DTIC-induced activation of genes involved in the immune response and leukocyte stimulation in patients treated with combined chemo-immunotherapy. In particular, it was found a progressive widening of T-cell receptor (TCR) repertoire diversity [58], accompanied by high avidity and high anti-tumor T-cell polyfunctionality [59]. This clinical finding does not appear to be in contrast with the well documented immunosuppressive activity of DTIC previously described, since there is a difference in the dose mainly used in the mouse model (i.e. 100 mg/Kg daily for 5 days) and in the clinical investigation (one single administration of 800 mg/sqm, toxicologically equivalent to 21 mg/Kg in man and 260 mg/kg in mouse [60]).

Adoptive immunotherapy studies in mice showed that transfer of T lymphocytes presensitized against xenogenized leukemia cells can prolong significantly the survival of immunodepressed (i.e. treated with 4 Gy total-body irradiation) recipients bearing the same xenogenized cells [61]. Of particular interest is the finding that a similar effect was attained in mouse brain after intracerebral challenge with xenogenized leukemia followed by intracerebral inoculation with Ag-specific CTL [62].

## ICPI AND CANCER IMMUNOTHERAPY

In the last 5 years we assisted to the steep increase of clinical investigations on cancer immunotherapy [63] - especially with the adoption of ICpI [64] - starting with supposedly immunogenic malignant diseases, such as melanoma and kidney carcinoma. Actually, the first clinical report on the therapeutic and toxic effects of the earliest ICpI utilized in cancer patients, i.e. Ipilimumab, can be traced back in 2003 when Hodi *et al*. [65] published their studies on this anti-CTLA-4 mAb in a clinical setting. A relatively long period of time was taken in the attempts to establish the best effective dose and schedule, and the safety of ICpI in a limited number of patients, mainly affected by metastatic melanoma. Thereafter, clinical studies involving ICpI-based immunotherapy were extended to a large variety of neoplastic diseases, as summarized in Tables 1 to 5 that illustrate the present state of the art in this area.

**Table 1:** Approval status and clinical development of immune checkpoint inhibitors

Agent	Characteristics	Clinical studies leading to drug approval and current status of clinical development relative to histological tumor type		FDA Approval
*Anti-CTLA-4 agents*				
Ipilimumab	Fully human anti-CTLA-4 IgG1k mAb	Melanoma	In a phase III trial on pretreated pts, median OS was significantly improved in the ipilimumab group (10.1 months *vs* 6.4 months in the control group) [224]. In another phase III trial, OS was better in the ipilimumab+dacarbazine group than in the dacarbazine group [225]. In phase II-III trials durable responses and prolonged survival have been reported [226]. In a phase III trial, median RFS was 26.1 months (95% CI 19.3-39.3) in the ipilimumab group *vs* 17.1 months (95% CI 13.4-21.6) in the placebo group (HR 0.75; 95% CI 0.64-0.90; p=0.0013); 3-year RFS was 46.5% (95% CI 41.5-51.3) in the ipilimumab group *vs* 34.8% (95% CI 30.1-39.5) in the placebo group [227].	∙ In patients with unresectable or metastatic melanoma (2011). ∙ As adjuvant therapy for patients with stage III melanoma (2015).
		NSCLC	Under evaluation in phase II/III clinical trials.	Pending
		SCLC, prostate cancer	Under evaluation in phase II clinical trials.	Pending
Tremelimumab	Fully human anti-CTLA-4 IgG2 mAb	Melanoma	In a phase III trial, median OS was 12.6 months (95% CI 10.8-14.3) for tremelimumab and 10.7 months (95% CI 9.36-11.96) for chemotherapy (HR, 0.88; p=0.127). ORRs were similar in the two arms, but response duration was significantly longer after tremelimumab (35.8 *vs* 13.7 months; p=0.0011) [228].	Not presented for approval.
		HCC, NSCLC	Under evaluation in phase II clinical trials	Pending
		Various cancer types	Under evaluation in phase I/II clinical trials	Pending

**Table 2:** Approval status and clinical development of immune checkpoint inhibitors

Agent	Characteristics	Clinical studies leading to drug approval and current status of clinical development relative to histological tumor type		FDA Approval
*Anti-PD-1 agents*				
Nivolumab	Fully human anti-PD-1 IgG4k mAb	Melanoma	· In a phase III trial, ORR was 31.7% (95% CI 23.5-40.8) in the nivolumab group *vs* 10.6% (95% CI 3.5-23.1) in the ICC group [229]. In a phase III trial, 1 year-OS was 72.9% (95% CI 65.5-78.9) in the nivolumab group *vs* 42.1% (95% CI 33.0-50.9) in the dacarbazine group (HR for death, 0.42; 99.79% CI 0.25-0.73; *p* < 0.001) [230]. In a phase III trial, median PFS was 11.5 months (95% CI 8.9-16.7) with nivolumab+ipilimumab, *vs* 2.9 months (95% CI 2.8-3.4) with ipilimumab (HR for death or disease progression, 0.42; 99.5% CI 0.31-0.57; *p* < 0.001), and 6.9 months (95% CI 4.3-9.5) with nivolumab (HR for the comparison with ipilimumab, 0.57; 99.5% CI 0.43-0.76; *p* < 0.001) [231].	∙ In patients with unresectable or metastatic melanoma who no longer respond to other drugs (2014). ∙ In combination with ipilimumab for the treatment of patients with BRAF V600 wild-type and BRAF V600 mutation-positive unresectable or metastatic melanoma (2015, 2016).
		NSCLC	In a phase III trial (on squamous-NSCLC patients), median OS was 9.2 months (95% CI 7.3-13.3) with nivolumab *vs* 6.0 months (95% CI 5.1-7.3) with docetaxel. The risk of death was 41% lower with nivolumab than with docetaxel (HR 0.59; 95% CI 0.44-0.79; *p* < 0.001). At 1 year, the OS rate was 42% (95% CI 34-50) with nivolumab *vs* 24% (95% CI 17-31) with docetaxel [232]. In a phase III trial (on nonsquamous-NSCLC patients), median OS was 12.2 months (95% CI 9.7-15.0) in the nivolumab group and 9.4 months (95% CI 8.1-10.7) in the docetaxel group (HR for death 0.73; 96% CI 0.59-0.89; *p* = 0.002). The 18-months OS rate was 39% (95% CI 34-45) with nivolumab *vs* 23% (95% CI 19-28) with docetaxel [233].	∙ In patients with metastatic NSCLC with progression on or after platinum-based chemotherapy. Patients with EGFR or ALK genomic tumor aberrations should have disease progression on FDA-approved therapy for these aberrations prior to receiving nivolumab (2015).
		RCC	In a phase III trial, median OS was 25.0 months (95% CI 21.8-not estimable) with nivolumab and 19.6 months (95% CI 17.6-23.1) with everolimus. HR for death with nivolumab *vs* everolimus was 0.73 (98.5% CI 0.57-0.93; *p* = 0.002) [234].	∙ In patients with metastatic RCC who have progressed on an anti-angiogenic agent (2015).

**Table 3:** Approval status and clinical development of immune checkpoint inhibitors

Agent	Characteristics	Clinical studies leading to drug approval and current status of clinical development relative to histological tumor type		FDA Approval
*Anti-PD-1 agents*				
Nivolumab	Fully human anti-PD-1 IgG4k mAb	HL	ORR was 87% (with 13% of patients having stable disease). PFS at 24 weeks was 86% [235].	∙ In patients with classical HL who have relapsed or progressed after autologous hematopoietic stem cell transplantation and post-transplantation brentuximab vedotin (2016).
		Gastric cancer, glioblastoma, SCCHN	Under evaluation in phase III clinical trials	Pending
		Anal cancer, AML, cervical cancer, NHL, nasopharinx carcinoma, pancreatic cancer	Under evaluation in phase II clinical trials	Pending
Pembrolizumab	Humanized anti-PD-1 IgG4k mAb	Melanoma	In a phase III trial, estimated 12-month survival rates were 74.1% in the group who received pembrolizumab 10 mg/Kg every 2 weeks, 68.4% in the group who received pembrolizumab 10 mg/Kg every 3 weeks, and 58.2% in the group who received 4 doses of 3 mg/Kg ipilimumab every 3 weeks (HR for death for pembrolizumab every 2 weeks, 0.63; 95% CI 0.47-0.83; *p* = 0.0005; and for pembrolizumab every 3 weeks, 0.69; 95% CI 0.52-0.90; *p* = 0.0036) [236].	∙ In melanoma patients following treatment with ipilimumab, or after treatment with ipilimumab and a BRAF inhibitor (2014).
		NSCLC	·In a phase III trial (KEYNOTE-010), median OS was 10.4 months with pembrolizumab 2 mg/kg, 12.7 months with pembrolizumab 10 mg/kg, and 8.5 months with docetaxel. OS was significantly longer for pembrolizumab 2 mg/kg *vs* docetaxel (HR 0.71, 95% CI 0.58-0.88; *p* = 0.0008) and for pembrolizumab 10 mg/kg *vs* docetaxel (0.61, 0.49-0.75; *p* < 0.0001) [237].	∙ In patients affected by metastatic NSCLC expressing PD-L1 and who have failed treatment with other chemotherapeutic agents (2015)

**Table 4:** Approval status and clinical development of immune checkpoint inhibitors

Agent	Characteristics	Clinical studies leading to drug approval and current status of clinical development relative to histological tumor type		FDA Approval
*Anti-PD-1 agents*				
Pembrolizumab	Humanized anti-PD-1 IgG4k mAb	NSCLC	In another open label phase III trial (KEYNOTE-024: first line pembrolizumab at a fixed dose of 200 mg every 3 weeks *vs* CDDP-based CT) median PFS was 10.3 months (95% CI 6.7 to not reached) in the pembrolizumab group *vs* 6.0 months (95% CI 4.2-6.2) in the CT group (HR for disease progression or death 0.50; 95% CI 0.37-0.68; *p* < 0.001). The estimated rate of OS at 6 months was 80.2% in the pembrolizumab group *vs* 72.4% in the CT (HR for death 0.60; 95% CI 0.41-0.89; *p* = 0.005) [238].	
		Gastric/GEJ cancer, SCCHN, urothelial cancer	Under evaluation in phase III clinical trials	Pending
		CRC, glioblastoma, HL, NHL, Merkel cell carcinoma, 3-BC	Under evaluation in phase II clinical trials	Pending
Pidilizumab	Humanized anti-PD-1 IgG1k mAb	Multiple myeloma, pancreatic and prostate cancer, RCC, sarcoma, thymic cancer, NHL	Under evaluation in phase II clinical trials	Pending

**Table 5:** Approval status and clinical development of immune checkpoint inhibitors

Agent	Characteristics	Clinical studies leading to drug approval and current status of clinical development relative to histological tumor type		FDA Approval
*Anti-PD-L1 agents*				
BMS-936559	A fully human anti-PD-L1 IgG4 mAb	NSCLC, RCC, CRC, melanoma	Under evaluation in phase I clinical trials	Pending
Atezolizumab (MPDL3280A)	Human anti-PD-L1 IgG1 mAb	Bladder cancer, NSCLC	Under evaluation in phase III clinical trials	Pending
		RCC	Under evaluation in phase II clinical trials	Pending
		CRC	Under evaluation in phase IIb clinical trials	Pending
		Melanoma	Under evaluation in phase I/II clinical trials	Pending
		Various cancer types	Under evaluation in phase I clinical trials	Pending
Durvalumab (MEDI4736)	Fully human anti-PD-L1 IgG1k mAb	NSCLC, SCCHN, CRC	Under evaluation in phase III clinical trials	Pending
		Various cancer types	Under evaluation in phase I clinical trials	Pending
Avelumab (MSB0019718C)	Fully human anti-PD-L1 IgG1 mAb	RCC, NSCLC	Under evaluation in phase III clinical trials	Pending
		Bladder, gastric and ovarian cancer, HNC, mesothelioma	Under evaluation in phase II clinical trials	Pending

3-BC, triple negative breast cancer; CDDP, cisplatin; CI, confidential interval; CRC, colorectal cancer; CT, chemotherapy; HCC, hepatocellular carcinoma; GEJ, gastroesophageal junction; HL, Hodgkin lymphoma; HNC, head and neck cancer; HR, hazard ratio; ICC, investigator's choice of chemotherapy; mAb, monoclonal antibody; NHL, non-Hodgkin lymphoma; NSCLC, non-small cell lung cancer; PD-1, programmed death 1; PD-L1, programmed death-ligand 1; ORR, overall response rate; PFS, progression-free survival; RCC, renal cell carcinoma; SCCHN, squamous cell cancer of head and neck; OS, overall survival; RFS, relapse-free survival; *vs*, *versus*.

## WHY IMMUNE CHECKPOINTS AS TARGET OF CANCER IMMUNOTHERAPY?

A detailed comprehension of the mechanisms involved in the antitumor immune response is essential for explaining the development of innovative therapeutic strategies based on ICpI. The generation of an effective antitumor T-cell response involves the passage through different steps including Ag-specific T-cell priming, differentiation, trafficking and killing of tumor cells by cytotoxic CD8^+^ T cells [66]. Furthermore, the amplitude and the quality of Ag-specific T-cell activation is a finely regulated process where the balance between TCR engagement and co-stimulatory as well as inhibitory signals is critical in order to maintain the self-tolerance and prevent T-cell over-activation [67]. Co-stimulatory molecules play a critical role during T-cell activation by inducing cytokine production and promoting T-cell proliferation [68] and are essential for lowering the threshold of TCR activation, supporting the responses against low-affinity Ags [69]. Poorly functional, differentiated CD8^+^ and CD4^+^ T cells are characterized by the loss of co-stimulatory molecules such as CD27 and CD28, which have been described also as useful markers for defining T-cell subpopulations at various stages of differentiation [70–73]. However a substantial redundancy in co-stimulatory receptor usage has been demonstrated and co-stimulation through inducible T-cell co-stimulator (a ICOS CD28 family member), CD137 and CD134 (members of the tumor necrosis factor family) contributes to enhance T-cell activation [73–77] in CD28^-^ T cells [73]. Therefore, additional changes to the loss of co-stimulatory receptors are involved in T-cell differentiation-related dysfunction. One such change is a rise in the expression of co-inhibitory receptors, commonly referred to as immune checkpoints, which negatively modulate the response of T cells to self proteins, chronic infection and tumor Ags and have been extensively studied over the last two decades.

## THE NUMBER OF IMMUNE CHECKPOINT PROTEINS IS UNEXPECTEDLY HIGH

Co-inhibitory receptors including Programmed Death-1 (PD-1), CTLA-4, Lymphocyte-Activation Gene since LAG-3 is acronim of Lymphocyte-Activation Gene 3 (LAG-3), T cell immunoglobulin mucin (TIM-3) and other molecules - some of which are described in the following paragraphs - are gradually up-regulated in T cells as they progress towards the acquisition of an “exhausted” phenotype. This phenotype is characterized by decreased production of effector cytokines and severely impaired anti-tumor and anti-viral functionality [78–80]. Tumors are capable of exploiting the homeostatic mechanisms regulated by these checkpoint molecules, affecting the immune system through several strategies including alterations in Ag expression and interference with T-cell priming and activation. Therefore, in addition to other regulatory effects on the surrounding microenvironment, the tumors escape immune-mediated detection and eradication [81].

## CANCER CELL DEFENSES AGAINST HOST'S IMMUNITY

Although effector T cells are able to enter the tumor micro-environment (TME), once there, they are negatively regulated by the cancer cells themselves and by immunosuppressive factors and infiltrating cells. Among these immunosuppressive elements are transforming growth factor-β, IL-6, vascular endothelial growth factor, Treg, [82] and myeloid-derived suppressor cells (MDSCs, [83–84]). In addition, potential antitumor T cells present in TME are exposed to checkpoint pathways which play a critical role in regulating T-cell phenotype and function [85–89]. Then, the immunosuppressive nature of TME drives the infiltrating tumor-specific T cells towards terminal differentiation and exhaustion [90]. Exhausted T cells lose their functional activity in terms of cytokine production in a hierarchical way: IL-2 production is lost first, tumor necrosis factor-α production at the intermediate phase, while interferon (IFN)-γ and granzyme B are lost at an advanced stage of exhaustion [91]. PD-1 is one of the major regulator of T-cell exhaustion, however immune checkpoint receptors operate by using rather distinctive and non-redundant signaling pathways. Moreover, co-inhibitory receptors have been shown to function synergistically, mostly in concert with PD-1, suggesting that distinct molecules provide individual functions able to impair T-cell responses [92–95].

## THE CTLA-4 PROTEIN

CTLA-4, the first immune checkpoint receptor to be clinically targeted, primarily controls the amplitude of T-cell activation, contributing to the preservation of T-cell homeostasis [5, 67]. CTLA-4 knockout mice die within three weeks from immune destruction of multiple organs [96], which proves a critical role as regulator of T-cell-dependent immune responses. CTLA-4 mainly antagonizes the activity of CD28 by sharing its ligands, CD80 and CD86 - expressed by Antigen Presenting Cells (APCs) - with a much higher overall affinity for both molecules compared to the contender co-stimulatory receptor [97]. Thus CTLA-4 expression reduces T-cell activity by competing with CD28 in the binding with CD80 and CD86 and by delivering inhibitory signals to the T cell [98–100]. Although CTLA-4 is also expressed by activated CD8^+^ effector T cells, this molecule plays its main physiologic role by down-regulating CD4^+^ helper T-cell activity. Importantly, CTLA-4 engagement improves the suppressive activity of Tregs [101, 102] which are typically concentrated in tumor tissues where they contribute to the inhibition of anti-tumor effector responses. It follows that CTLA-4 blockade results in a wide enhancement of the immune responses dependent on CD4^+^ helper T cells. The expression of CTLA-4 is constitutive on Tregs, where it represents a target gene of the forkhead transcription factor Foxp3 [103, 104], a major player in the development and function of Tregs. The critical function played by CTLA-4 in regulating Treg function is confirmed by the observation that mice with CTLA-4-lacking Tregs show systemic lympho-proliferation, fatal T cell-mediated autoimmune diseases and potent anti-tumor immunity.

The engagement of CTLA-4 has been shown to inhibit CD3/CD28-mediated activation of the serine/threonine kinase AKT in T cells mainly through the recruitment and activation of the protein phosphatase 2A [105, 106] and not through phosphatidylinositol 3-kinase (PI3K) inactivation. However, CTLA-4 has been reported to bind also the tyrosine phosphatase SHP-2. SHP-2 activation suppresses the CD3/CD28-induced T-cell transcriptional profile and results in the inactivation of LCK and ZAP-70 kinases as well as the dephosphorylation of the CD3-ζ chains of the TCR complex, thus reducing activation of the TCR signaling [105, 106]. However, the role of SHP-2 in the CTLA-4 negative regulation T-cell function has not been fully elucidated yet.

The intrinsic inhibitory mechanisms mediated by the intracellular domain of CTLA-4 are accompanied by a cell-extrinsic mechanism which involves the capture of CD80 and CD86 from APCs by trans-endocytosis, the degradation of these molecules inside the T cell and the inhibition of T-cell activation through the CD28 engagement [107].

## THE PD-1 PROTEIN AND ITS LIGANDS

The inhibitory mechanisms triggered by PD-1 are distinct from those described for CTLA-4. The relatively milder and more chronic clinical pathological phenotypes which result from PD-1 blockade are probably related to the cell-intrinsic function and to the regulation of PD-1 expression.

PD-1 is a member of the CD28 superfamily, and plays its physiologic inhibitory role by regulating the induction and maintenance of peripheral tolerance, thus protecting tissues from autoimmunity, especially during inflammatory and Ag-specific responses [108–111, 67]. In particular, PD-1 exerts its inhibitory functions only after T-cell activation, following Ag recognition, thus contributing to restrain the effector phase of T-cell mediated tumor rejection [112]. The amount and source of Ag determines the strength and the kinetics of T-cell activation as well as the extent and regulation of PD-1 expression. Moreover, the ligands of PD-1, able to activate the molecule on T cells, are expressed to a different extent depending on the cell type [109].

PD-1 is expressed on activated T cells as well as on B and natural killer (NK) cells, activated monocytes and some subsets of dendritic cells (DCs) [109], implying a broad contribution in the immune regulation. The two ligands for PD-1 are PD-L1 (B7-H1, CD274) and PD-L2 (B7-DC, CD273) [109, 113–115]. Moreover, it has been unexpectedly observed a molecular interaction between PD-L1 and CD80, which indicates that CD80 expressed on T cells and APCs can possibly work as a receptor rather than a ligand, eliciting inhibitory signals when engaged by PD-L1 [116].

PD-L1 is expressed on resting T cells, B cells, DCs, macrophages, vascular endothelial cells, pancreatic islet cells and in various types of cancers, including Non Small Cell Lung Cancer (NSCLC), melanoma, renal cell carcinoma, gastric cancer, hepatocellular carcinoma as well as cutaneous lymphoma, multiple myeloma and various leukemias [117–125]. Elevated expression of PD-L1 has been found to represent an adverse prognostic biomarker in NSCLC [126]. PD-L1 can be up-regulated by IFN-γ produced by tumor-infiltrating T cells thus promoting the progression of cancer [127]. Moreover, *in vitro* exposure of human lymphoma cells to T cells or monocytes has been shown to up-regulate blast-associated PD-L1 expression [128]. Also radiation or chemotherapy can up-regulate PD-L1 [129, 130]. In particular, radiotherapy may induce direct killing of tumor cells and multiple immune-modulatory changes that can potentially influence the effectiveness of immunotherapy [129, 131]. On the other hand, NSCLC patients are significantly more responsive to anti-PD-1 mAbs when malignant cells over-express PD-L1 [132].

PD-L2, although less expressed by tumors than PD-L1, also binds to PD-1 and regulates T-cell function [133]. PD-L2 is constitutively expressed at low levels but can be induced on DCs, macrophages and mast cells in response to IL-4 and type 1 IFNs [134]. These differences in the pattern of expression suggest different functions played by the engagement of PD-1 in the immune regulation within distinct cell contexts. The limited expression of PD-L2 primarily to APC reflects a function in the control of the T-cell priming, while the wide occurrence of PD-L1 speaks in favor of an overall protective function of peripheral tissues from extreme inflammation.

Viruses and tumors exploit the negative regulatory function of PD-1, causing the inhibition of effector T-cell functionality, which translates into the onset of chronic infections and represents a major mechanism of immune resistance within the TME favoring the tumor progression.

Upon stimulation, PD-1, through its association with the SHP2 phosphatase, inhibits the proximal TCR signaling, leading to a strong reduction of T-cell functionality. PD-1 also prevents AKT phosphorylation by inhibiting CD28-mediated stimulation of PI3K [105, 106]. Therefore, CTLA-4 and PD-1 inhibit AKT by distinctive mechanisms.

Of remarkable importance is the finding that PD-1 blocks T-cell cycle progression through the G_1_ phase by suppressing the transcription of SKP2, a factor encoding a component of the ubiquitin ligase SCF^Skp2^ able to degrade p27^kip1^, an inhibitor of cyclin-dependent kinases [135]. PD-1-mediated decrease of SKP2 transcription is achieved through the inhibition of PI3K/AKT, Ras and extracellular signal-regulated kinase (ERK) signaling. IL-2 partially restores SKP2 expression, probably through the activation of ERK, but not the AKT signaling, demonstrating that PD-1 is able to impair T-cell proliferative potential by affecting multiple regulators of the cell cycle.

T-cell exhaustion is related to physical T-cell depletion especially in cancer. Apoptosis is one of the several potential mechanisms involved in PD-L1-associated T-cell death, which is supported by the inverse correlation existing between PD-L1 expression in tumor tissues and the number of tumor infiltrating lymphocytes (TILs) [136].

Foxp3^+^ Tregs express PD-1 and PD-L1 [137] and a critical function for the PD-1 engagement in the generation of Tregs has been clearly demonstrated [138]. In particular, the engagement of PD-1 on naive T cells can lead to the development of induced Treg cells partly through the inhibition of AKT/mTOR signaling [139]. Being many tumors highly infiltrated with Tregs which further suppress T-cell effector responses, PD-1 blockade can potentially enhance the antitumor responses also by decreasing the amount and the suppressive action of intra-tumoral Tregs.

The inhibitory function of the PD-1/PD-L1 engagement plays a critical role in reducing the immune-surveillance against tumors by inducing T-cell exhaustion. Therefore, the PD-1/PD-L1 pathway has become an attractive therapeutic target in the setting of cancer. PD-1 is highly expressed on exhausted T cells that develop in the setting of chronic Ag stimulation such as cancer and blockade of PD-L1 or PD-1 can reinvigorate the function of exhausted T cells. Blockade of PD-L1/PD-1 has been extensively shown to enhance T-cell anti-tumor function [140] including immune responses affecting malignant cell growth in the brain [141]. Interestingly, while the PD-L1/PD-1 signaling pathway is abundantly engaged in the TME, expression of PD-1 on peripheral CD4^+^ and CD8^+^ T cells has been shown to increase with tumor progression [142].

Several clinical studies have shown that high expression of PD-1 ligands on tumors correlates with poor prognosis [126, 143], which strongly suggests that the engagement of PD-L1/PD-1 pathway supports tumor escape from antitumor T-cell control. However, although co-inhibitory receptors have so far been considered to mark terminally differentiated “exhausted” T cells, they have recently been also associated with the activation status and the differentiation profile of Ag-specific T cells [59, 144] and must be considered instrumental for limiting the self-tissue damage at the tumor site. As stated before, the fate of T cells after the encounter with the specific Ag is determined also by additional inputs through co-receptors which finely regulate strength, duration and properties of the response upon interaction with their ligands. Co-expression of PD-1 with other co-stimulatory or co-inhibitory molecules in a particular Ag-context may then represent a rheostat in the control of highly reactive stimulated T cells [59, 145].

## THE KILLER-CELL LECTIN LIKE RECEPTOR G1 (KLRG1)

The expression of KLRG1 on Ag-experienced T cells and NK cells increases considerably with age and differentiation [146–148], with the highest expression observed in memory and in end-stage differentiated T cells [149]. An inhibitory role for KLRG1 on the cytolytic activity of polyclonal human NK cells has been suggested [150]. Moreover a critical role has been demonstrated for KLRG1 in the inhibition of AKT phosphorylation at the ser473 site, which results in a compromised proliferation of primary CD8^+^ T cells [148]. Actually, the loss of the ability to phosphorylate AKT at ser473 is a functional alteration that occurs throughout progressive T-cell differentiation and ageing [73, 151].

## THE LAG-3 PROTEIN

LAG-3 belongs to the immunoglobulin superfamily and is a crucial regulator of T-cell function [92, 152]. It is expressed on activated T cells, B cells, NK cells, DCs and TILs, but not on resting T cells [153, 154].

LAG-3 associates with the CD3-TCR complex after the TCR engagement and negatively regulates the TCR signaling in effector T cells in a pattern similar to that observed for CTLA-4 [155], impairing T-cell proliferation, homeostasis and functionality through the inhibition of calcium fluxes (156]. Similar to CD4 molecule, LAG-3 oligomerizes at the surface of T cells and binds to MHC class II molecules on APCs but with significantly higher affinity as compared with the CD4 molecule [157], thus decreasing the Ag-dependent stimulation of CD4^+^ T cells. However, a double role in regulating T-cell functionality has been suggested by the observation that expression of LAG-3 on T cells is associated with either the down-regulation of cytokine secretion [158, 159] or the induction of Th1 cytokine production, including IL-2 [160]. LAG-3 plays also a critical role in the control of the functional activity of both natural and induced immunosuppressive Tregs. As described for CTLA-4 and PD-1, LAG-3 is essential for the extrinsic regulation of Treg homeostasis and development [161]. A population of expanded CD4^+^CD25^high^Foxp3^+^ Tregs expressing LAG-3 has been identified in the peripheral blood, in T cells of tumor-invaded lymph nodes and in T cells infiltrating the visceral metastasis of melanoma and sarcoma patients [162]. Of particular interest is the observation that high LAG-3-expression makes effector T cells more susceptible to Treg-mediated suppression [154].

LAG-3 is highly co-expressed with other immune inhibitory molecules, including TIM-3, PD-1, 2B4 and T-cell immunoglobulin and ITIM domain (TIGIT), on tumor-specific CD4^+^ effector T cells [163]. On Melan-A specific CD8^+^ T cells isolated from melanoma patients, LAG-3 is highly co-expressed with PD-1 and TIM-3 [59]. LAG-3 itself can directly modulate the activity of PD-1^+^ T cells [163] and the synergy between LAG-3 and PD-1 can potentiate the tumor-induced tolerance [164].

## TIM-3 PROTEIN

TIM-3 is a glycoprotein which possesses on its extracellular portion both the immunoglobulin and the mucin domain. TIM-3 has been initially observed on terminally differentiated IFNγ-producing CD4^+^ Th1 cells and CD8^+^ cytotoxic T cells [165, 166], as well as on Th17, DCs, monocytes, Tregs, mast cells, NK cells, TILs and tumor cells, including melanoma, squamous cell carcinoma, gastric cancer and NSCLC cells, but not on CD4^+^ Th2 cells [154].

The most important role of TIM-3 is the negative control of Th1 immunity and the induction of peripheral tolerance [167]. The TIM-3/galectin-9 pathway engagement reduces the proliferative potential and functionality of Th1 and Th17 cells [168] and contributes to the immune-suppressive environment of TME through the promotion of Treg development [169]. TIM-3 also provides a new surface marker able to describe activated tumor infiltrating Tregs, which have been found to co-express higher levels of PD-1, CTLA-4 and the glucocorticoid-induced tumour necrosis factor receptor-related protein [170].

Consistent with the observation that the TIM-3 and PD-1 expressing CD8^+^ T cells represent the most exhausted TIL population, co-blockade of the TIM-3 and PD-1 signaling pathways has been shown to induce a more vigorous antitumor outcome as compared with PD-1 blockade alone [171].

## THE TIGIT RECEPTOR

The co-inhibitory receptor TIGIT, that contains the immunoreceptor tyrosine-based inhibitory motif (ITIM), has been initially described as a modest inhibitor of CD4^+^ T cell priming and NK cell killing activity. More recently, it has been shown to be highly expressed by tumor-infiltrating CD8^+^ T cells in parallel with PD-1 [172], as well as in models of chronic viral infection. In particular, TIGIT has been proven to be a critical and specific regulator of CD8^+^ T cell-dependent chronic immune responses. Co-blockade of TIGIT and PD-L1 improves synergistically the CD8^+^ T-cell effector functional activity [173], an effect abolished by blockade of the TIGIT's corresponding co-stimulatory receptor, CD226. The hypothesis that TIGIT may represent a critical collaborator of the PD-1/PD-L1 engagement in order to limit the activity of chronically stimulated CD8^+^ T cells is supported by the observation that co-blockade of TIGIT and PD-1 is essential to restore the anti-tumor functional activity of effector CD8^+^ T cells within the highly immunosuppressive TME.

TIGIT has been shown to identify the most dysfunctional subset of effector CD8^+^ T cells in tumors, and the tumor-infiltrating Tregs characterized by a highly suppressive phenotype. TIGIT signaling controls Treg phenotype, and Tregs show an increased expression of TIM-3 in the tumor tissue, where TIM-3 and TIGIT synergize in the suppression of the immune responses [174]. TIGIT expressing Foxp3^+^ Tregs have been recently identified as a subset able to specifically suppress pro-inflammatory Th1 and Th17, but not Th2 cell responses [175].

## THE NK CELL RECEPTOR 2B4

2B4 belongs to the CD2 family and is expressed on NK, γ/δ and memory CD8^+^ T cells [176]. The murine NK receptor 2B4 displays both inhibitory and activating functions, whereas human 2B4 has been reported to be mainly an activating molecule. In what way murine 2B4 can act both as an activating and inhibitory molecule and what distinguishes its function from human 2B4 receptor is still under investigation [177]. The amount of 2B4 expression and the level of 2B4 cross-linking play a significant role in the regulation of 2B4-mediated T-cell signaling pathway. A substantial reduction of T-cell activation has been observed with high levels and a strong engagement of 2B4, in the presence of a weak activation of T-cell signaling-associated molecules. Therefore 2B4 can have opposite effects depending on the degree of receptor expression, the level of its ligation, and the relative abundance of selected adaptor molecules [176].

## THE B AND T LYMPHOCYTE ATTENUATOR (BTLA)

This molecule has been identified as another co-inhibitory receptor with structural and functional similarities to CTLA-4 and PD-1 [178]. BTLA is induced during T-cell activation and remains expressed on Th1 but not on Th2 CD4^+^ T cells. The engagement of BTLA induces its tyrosine phosphorylation and association with the tyrosine phosphatases SHP-1 and SHP-2, thus attenuating, through the subsequent inhibition of TCR activation, the production of IL-2. BTLA is also expressed on activated CD8^+^ T cells where it induces functional inhibition through its ligand herpes virus entry mediator (HVEM). In human virus Ag-specific CD8^+^ T cells the expression of BTLA is gradually down-regulated as they differentiate into effector cells [179]. In contrast, human melanoma Ag-specific effector CD8^+^ T cells persistently express high levels of BTLA and remain susceptible to the functional inhibition mediated by its ligand HVEM. Such persistence of BTLA expression has also been found in tumor Ag-specific CD8^+^ T cells isolated from melanoma patients with spontaneous anti-tumor immune responses and after peptide vaccination. Interestingly, the co-expression of BTLA with PD-1 and TIM-3 has been shown to identify the most dysfunctional NY-ESO-1-specific CD8^+^ T cell population in melanoma patients [80].

## THE INDOLEAMINE 2,3 DIOXYGENASE (IDO)

The control of the access to nutrients is an important strategy able to regulate cellular responses to proliferative stimuli. The IDO enzyme is responsible for a critical step in the metabolic pathway that converts the essential amino acid L-tryptophan into L-kynurenine, and has been shown to exert a highly suppressive activity on T cells [180]. Both L-tryptophan depletion and L-kynurenine accumulation appear implicated in the immunosuppressive activity of IDO [180].

IDO is expressed in different cell types, including DCs and macrophages and plays a critical role in immunological tolerance. Several cancer types can express themselves IDO or induce its expression in host APCs, either directly or indirectly, thus leading to the impairment of T-cell functionality [181]. Tumors are then able to create an immunosuppressive microenvironment able to impair T-cell mediated antitumor immune response by inducing IDO over-expression [182]. Interestingly, a tumor-mediated IDO-dependent activation of suppressive Tregs has also been documented [183].

In a mouse model, IDO expressed by the host immune cells has been shown to reduce the infiltration of tumor-reactive T cells in B16 tumors, inducing resistance to immunotherapy with mAbs targeting CTLA-4 and PD-1 [184]. Differently, IDO-KO mice have been shown to mount a good anti-tumor response following treatment with anti-CTLA-4 mAbs. This IDO-induced resistance to T-cell-targeting immunotherapies has been associated with a general increase in the recruitment of MDSCs into the TME. A comparable association between IDO expression and MDSC infiltration into the TME has been observed in human melanoma samples and animal tumor models naturally expressing high levels of IDO. These observations prove that IDO represents a key regulator of immunosuppression both at the systemic and TME level, and provide a strong rationale for therapeutic targeting of this pathway.

## CANCER IMMUNOEDITING

The paradigm of cancer immunoediting shows that, during the different phases of cancerogenesis and tumor progression, the immune response shapes the tumor with the selection of tumor variants that escape immune recognition [81, 185, 186].

One of the crucial challenges in immunology is the comprehension of the control of the immune system on cancer development and progression. The immune system plays a dual role in cancer development, being able to contrast tumor growth but also to promote progression either by selecting tumor cells resistant to the immunological control or by modeling conditions within the TME. An essential principle of cancer immunosurveillance is that cancer cells express a number of Ags different from their non-transformed counterparts. These Ags include differentiation, mutated, overexpressed, viral and cancer/testis molecules [186]. Changes capable of conferring resistance to the attack by the immune system include the loss of expression of tumor Ags and an altered expression pattern of class I molecules, as a consequence of critical deficiencies in the Ag processing pathway. This promotes a reduced expression or a total loss of class I peptide presentation, which in turn allows tumor cell escape from Ag-specific effector CD8^+^ T-cell killing.

In 2002 Dunn *et al*. [185] showed that tumors developed in the absence of an intact immune system (classified as “unedited” tumors) were more immunogenic than similar tumors derived from immunocompetent mice (classified as “edited” tumors). This observation demonstrated that the immune system controls tumors also in terms of quality and immunogenicity. The concept that the immune system, besides its protective role, also shapes tumor immunogenicity constitutes the starting point of the “cancer immunoediting hypothesis”. Cancer immunoediting consists of three sequential phases: elimination, equilibrium, and escape [186]. In the elimination phase, the immune system works at eliminating developing tumors before they become clinically apparent. Cancer immunoediting enters the equilibrium phase when cancer cells are not totally eliminated but cancer growth is prevented by immune-mediated mechanisms. In particular, the editing of tumor immunogenicity takes place during the equilibrium phase, when some tumor cells become not recognizable by the immune system (due to Ag loss or defects in Ag processing or presentation), or resistant to effector T-cells, or induce an immunosuppressive environment within the TME. These tumor cells may then enter the escape phase, where cancer growth is no longer inhibited by the immune system and the tumor becomes clinically apparent. However, external factors including the immune senescence associated with ageing may influence this directionality, and cancer cells may directly progress toward the escape phase (186].

## THE ROLE OF MUTATION-INDUCED TUMOR NEOANTIGENS AND SUSCEPTIBILITY TO ICPI

As tumors grow, they acquire mutations, some of which generate neoantigens potentially able to elicit host's immune responses [187]. In relatively recent years, a growing number of clinical studies has revealed a direct correlation between mutation load detectable in malignant cells and susceptibility to the therapeutic effectiveness of ICpI. In particular, a direct relationship between the extent of non-synonymous mutation burden and durable clinical benefit was found in NSCLC [188] and in ovarian cancer harboring mutated BRCA1/2 gene [189]. Moreover, Le *et al*. [190] demonstrated that pembrolizumab, a mAb able to suppress PD-1 function, was significantly more active against metastatic colorectal cancer when malignant cells were deficient for the MMR system. In this case, whole-exome sequencing showed that MMR-deficient tumor cells had a number of somatic mutations approximately 25 times higher than that detectable in MMR-proficient tumors.

Pharmacologically-driven non-synonymous somatic mutations leading to increased immunogenicity of tumors could be induced not only by triazenes but potentially also by other antitumor agents. Actually, cisplatin that was found to be highly mutagenic in a lymphoblastic cell line model [191] merits further investigation for possible DIX effects. In addition, drugs able to influence the epigenome profile of malignant cells can provide novel immunogenic targets through up-regulation of the expression of non-pathogenic retroviruses inserted in human cell genome (“viral mimicry”) as found for colorectal cancer [192] and supposedly applicable to neuroendocrine tumors [193].

The role of somatic mutations in the immunogenicity of neoplastic cells and its relevance not only in the therapeutic response to ICpI but also in vaccine development has been widely confirmed by several authors [194–198]. However, a note of caution stems from a clinical investigation on NSCLC reported by McGranahan [198] who stressed that, in spite of large neoepitope burden, an excess of neoantigen intratumor heterogeneity accompanied by the absence of common antigenic determinants in all malignant cells does not provide therapeutic advantage upon ICpI administration. The authors termed “clonal neoantigens” tumor-specific Ags - generally originated by mutational mechanisms - that are present in the whole tumor cell population. On the contrary, a number of different non cross-reacting tumor-associated neoantigens present in the bulk malignant cells were designated as “subclonal neoantigens”. Therefore, it is reasonable to predict that in case of clonal neoantigens, host's immune response can be efficiently directed against a common epitope present in all tumor cells. Conversely, in case of subclonal neoantigens, each of the great number of malignant cell-associated neoantigens does not reach the threshold required to elicit an effective cell-mediated response even if assisted by ICpI-based therapy.

## THERAPEUTIC POTENTIAL OF DIX IN APPROPRIATE COMBINATION WITH ICPI

In the last few years we assisted to the enormous expansion of preclinical and clinical studies on cancer immunotherapy based on tumor-associated neoantigens targeted by cytotoxic effector cells generated by immune mechanisms amplified by ICpI. Since, as previously stated, a large number of investigations found that tumor neoantigens are generally the result of somatic mutations, DIX appears to open new and exciting perspectives in the future development of ICpI-dependent cancer immunotherapy. In animal models, DIX was found to be inducible *in vivo* or *in vitro* in a number of mouse leukemia syngeneic with strains homozygous for different types of MHC, such as *H-2*d (e.g. L1210 [1, 12, 15, 18, 19, 36, 199], LSTRA [9], L5178Y [13], P815 [200, 201]) *H-2*b (RBL-5 [9]) and *H-2*k (K36 [202]) although in this case the immunogenicity of DTIC-treated cells was found to be modest. This observation implies that DIX is not limited to a specific genetic pattern in mouse models. However, it must be stressed that all these malignant cell types are characterized by complete homozygosity of the entire genome and we do not know the influence that this particular biological situation could have on the mutational profile induced by triazenes.

As previously mentioned in this report, the earliest hint of triazene-induced DIX in human neoplasias was obtained by D’Atri *et al.* in 1994 [11] who found that selected CD8^+^ CTL clones directed against *in vitro* triazene-treated H-125 human lung adenocarcinoma cell line were able to lyse the drug-treated cells but not the untreated parental cells.

Several difficulties must be overcome before formulating an adequate DIX-based design of cancer immunotherapy centered on ICpI administration to amplify host's cell-mediated responses targeting pharmacologically-induced tumor neoantigens. Firstly, somatic mutations affecting steps of the complex apparatus involved in Ag presentation by malignant cells could compromise its function [203, 204] thus subtracting target cells from lethal attack by effectors of host's immunity. One of the most utilized devices adopted by tumors to evade T-cell mediated immunity consists in down-regulation of MHC class I molecule expression thereby precluding non-self peptide presentation that can be recognized by cytotoxic T cells [203, 205]. Several approaches have been described to be able to increase MHC expression and to overcome defects in Ag presentation pathways. In particular, upregulation of HLA class I expression in malignant cells has been obtained using pharmacological agents such as DNA hypomethylating compounds (e.g. azacytidyne, [205, 206] or, more recently, SGI-110 [207]) and histone deacetylase inhibitors. Likewise, several studies indicated that histone deacetylase inhibition is able to increase consistently various mechanisms involved in Ag presentation, including, for example, DC function [208].

Unexpectedly, neoantigen pattern of each single patient could also provide additional problems concerning the efficiency of ICpI treatment. Several data from the literature point out that patients with tumors endowed with mutations that are predicted to be immunogenic, show survival benefit from ICpI administration [209]. However, as previously mentioned in this review, drug-induced generation of extremely high numbers of different neoantigens within the tumor cell population (i.e. “subclonal neoantigens” [198]) could be unable to elicit an adequate host's response. It is reasonable to predict that treatment with high-dose classical cytotoxic or targeted chemotherapy could reduce substantially the polyclonality of malignant cells pre-exposed to drug-induced mutagenization. It follows that a relatively low number of drug-resistant clones could provide an adequate antigenic stimulus to the host's immune apparatus. The findings described by Nicolin *et al.* [210], who found that several different L1210 cell lines resistant to a number of antitumor agents are limitedly but significantly immunogenic for the histocompatible host, could be interpreted on this basis.

As an example, a purely speculative therapeutic design stemming from a rational application of triazene-related DIX followed by ICpI should contain at least 3 sequential phases. The first step (i.e. DIX phase) includes temozolomide treatment in two types of patients, i.e. those bearing MGMT-deficient neoplasia, or those affected by MGMT-proficient malignancy. In the latter case, temozolomide-induced xenogenization is prevented by the efficient removal of methyl adducts at O^6^-guanine [211, 212] and the triazene compound must be associated with an MGMT inhibiting agent [213], including O^6^-benzylguanine [214, 215], cisplatin [216–218] or Lomeguatrib, that is the best drug available today [3, 47, 214, 219–221]. However, Lomeguatrib downregulates profoundly MGMT expression in both neoplastic and normal cells. Therefore, the dosage of temozolomide must be carefully controlled since this association is characterized by elevated myelotoxicity [221]. Alternatively, if MGMT inhibitors are not available, a sequential administration of high-dose triazenes that deplete target cells of MGMT followed by a DIX generating treatment with the same triazene could be taken into consideration [ 222]. During the DIX phase, it is possible that normal hematopoietic cells undergo malignant transformation. Actually, treatment with temozolomide, especially in brain tumor cases, has been found to be followed by the appearance of leukemia or myelodysplastic syndrome (reviewed in [223]). It can be suggested that rare clones of target hematopoietic cells survive temozolomide-induced apoptosis thank to MMR deficiency [47] or inadequacy of the apoptotic function. On the other hand, it is reasonable to hypothesize that these clones could be also endowed with at least a limited degree of immunogenicity that renders the cells susceptible to host's immune attack, especially after extreme amplification by ICpI treatment.

The second phase (“clonal simplification”) comes after the completion of DIX, and is directed to reduce the number of xenogenized tumor cell clones through treatment with a standard chemotherapy approach. During this phase it is quite possible that the number of immunogenic xenogenized subclones will be sensibly reduced, since a limited number of drug-resistant clones are selected. Therefore, at the end of phase 2 the host should be able to mount an even minimal immune response, although largely inefficient to control tumor growth.

The third phase (ICpI-immune amplification) should complete the work through treatment with ICpI, leading to immune-mediated suppression of target xenogenized malignant cells and bone-marrow-derived pre-malignant cells.

In conclusion, triazene-induced DIX could open up a new avenue in the area of tumor immunotherapy. Indeed, this treatment modality discloses the invaluable opportunity to design a pharmacological control of neoantigen generation that provides the molecular bases of efficient ICpI-dependent suppression of malignant cell growth.

## Abbreviations

Ag(s): antigen(s)
APC: antigen presenting cells
BCNU: bis-chloroethyl-nitrosourea
BTLA: B and T lymphocyte attenuator
CTL: cytotoxic T lymphocytes
CTLA-4: cytotoxic T lymphocyte antigen-4
CX: chemical xenogenization
DC: dendritic cells
DIX: drug-induced xenogenization
DTIC: dimethyltriazene-imidazole-4-carboxamide,
ERK: extracellular signal-regulated kinase
HVEM: herpes virus entry mediator
ICpI: immune checkpoint inhibitors
IDO: indoleamine 2,3 dioxygenase
IFN: interferon
IL: interleukin
ITIM: immunoreceptor tyrosine-based inhibitory motif
KLRG1: killer-cell lectin like receptor G1
LAG-3: lymphocyte activation gene-3
mAb: monoclonal antibody
MDSCs: myeloid-derived suppressor cells
MGMT: O6-methylguanine-DNA methyltransferase
MHC: major histocompatibility complex
MMR: mismatch repair
MNNG: N-methyl-N’-nitro-N-nitrosoguanidine
MST: median survival time
NK: natural killer
NSCLC: non small cell lung cancer
O^6^-MeG: O^6^-methylguanine
PD-1/2: programmed death-1/2
PD-L1/2: programmed death ligand 1/2
PI3K: phosphatidylinositol 3-kinase
TCR: T-cell receptor
TIGIT: T-cell immunoglobulin and ITIM domain
TIL: tumor infiltrating lymphocytes
TIM-3: T cell immunoglobulin mucin-3
TME: tumor micro-environment
Treg: regulatory T cells
